# Plasticity and modular control of locomotor patterns in neurological disorders with motor deficits

**DOI:** 10.3389/fncom.2013.00123

**Published:** 2013-09-10

**Authors:** Y. P. Ivanenko, G. Cappellini, I. A. Solopova, A. A. Grishin, M. J. MacLellan, R. E. Poppele, F. Lacquaniti

**Affiliations:** ^1^Laboratory of Neuromotor Physiology, Santa Lucia FoundationRome, Italy; ^2^Centre of Space Bio-Medicine, University of Rome Tor VergataRome, Italy; ^3^Laboratory of Neurobiology of Motor Control, Institute for Information Transmission Problems, Russian Academy of SciencesMoscow, Russia; ^4^Department of Neuroscience, University of MinnesotaMinneapolis, MN, USA; ^5^Department of Neuroscience, University of Rome Tor VergataRome, Italy

**Keywords:** compensation, plantarflexor weakness, spinal cord injury, stroke, EMG activity, modularity, locomotor pattern generation

## Abstract

Human locomotor movements exhibit considerable variability and are highly complex in terms of both neural activation and biomechanical output. The building blocks with which the central nervous system constructs these motor patterns can be preserved in patients with various sensory-motor disorders. In particular, several studies highlighted a modular burst-like organization of the muscle activity. Here we review and discuss this issue with a particular emphasis on the various examples of adaptation of locomotor patterns in patients (with large fiber neuropathy, amputees, stroke and spinal cord injury). The results highlight plasticity and different solutions to reorganize muscle patterns in both peripheral and central nervous system lesions. The findings are discussed in a general context of compensatory gait mechanisms, spatiotemporal architecture and modularity of the locomotor program.

## Introduction

Investigating locomotor responses after neurological lesions is fundamental to the development of improved rehabilitation strategies and to explore the mechanisms involved in improving locomotor function. The problem of motor neurorehabilitation is significant and complex. Numerous studies have shown that motor activity after brain damage plays an essential role in anatomo-physiological reorganization, which may occur in the areas adjacent to the damage (Cao et al., 1998; Nelles et al., 1999). Nevertheless, the building blocks with which the central nervous system constructs the motor patterns can be preserved in patients with neurological disorders. In particular, several studies highlighted a modular burst-like organization of muscle activity.

While biomechanical and neural aspects of human locomotion have been documented in many studies both in normal and pathological gait, the architecture of neural circuits and the nature of descending neural signals that are involved in locomotor control remain elusive in humans. To date, little work has been completed on characterizing the neural substrates for modularity in both healthy individuals and in neurological patients with different sensory-motor disorders. A number of studies explored the bases of central motor programming by decomposing muscle activation patterns as a means to look backward from the periphery to the CNS (Davis and Vaughan, 1993; Prentice et al., 1998; d'Avella and Bizzi, 2005; Ivanenko et al., 2006; Giszter et al., 2007; Tresch and Jarc, 2009; Chvatal and Ting, 2012; Bizzi and Cheung, 2013; Lacquaniti et al., 2013). While different studies use different decomposition techniques, the common message is the emphasis on modular architecture of the motor output. Furthermore, these computational techniques often converge to a similar solution (Ivanenko et al., 2005; Tresch et al., 2006). The data and concepts discussed here offer a new approach to characterizing the mechanisms underlying control of human locomotion that may potentially benefit the study of pathological gait and the ability of current therapeutic exercises to improve patient outcomes.

In patients, the mechanisms involved in locomotor improvements may rely on the inherent spatiotemporal organization of neural circuitry and its adaptability. The question arises as to whether the rhythmic patterning elements are invariant when muscle activation patterns can be compromised by spinal cord lesions, brain damage and other motor disturbances. Compensatory strategies for plantarflexor weakness or after distal limb segment amputation also represent important examples of locomotor adaptations. Several recent studies provide some clues on this topic. We will consider and discuss these examples in the following sections.

## Locomotor patterns in healthy subjects

**Figure 1:**
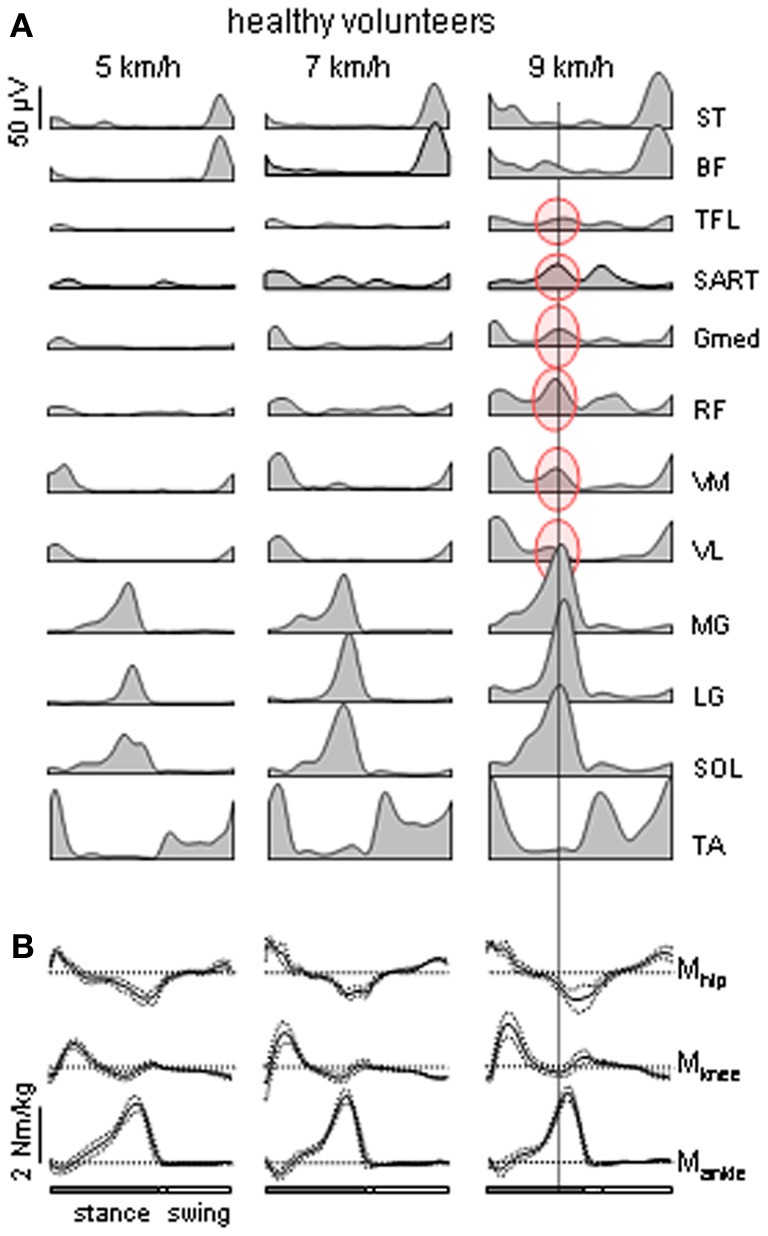
**Motor patterns in healthy volunteers. (A)** Ensemble-averaged EMGs (*n* = 8 subjects) recorded from 10 ipsilateral leg muscles during walking on a treadmill at 5, 7, and 9 km/h. At 9 km/h, there is an “atypical” burst of activity in several thigh muscles that is synchronous with the peak activity in the calf muscles [the data are illustrated from Ivanenko et al. (2008)]. ST, semitendinosus; BF, biceps femoris; TFL, tensor fascia latae; SART, Sartorius; Gmed, RF, rectus femoris; VM, vastus medialis; VL, vastus lateralis; MG, gastrocnemius medialis; LG, gastrocnemius lateralis; SOL, soleus; TA, tibialis anterior. **(B)** ensemble-averaged (±*SD*) ankle, knee and hip moments of force (normalized to the subject's weight) of the right leg during overground walking at about the same speeds (as in panel **A**) in one representative healthy subject.

Muscle activity during normal locomotion has both invariant and variant features. In each step, the control system needs to compensate for body weight, provide forward and lateral stability and maintain forward progression. The coordination of the musculoskeletal system with non-linear properties and multiple degrees of freedom is complex and requires activity of tens of leg muscles. Major muscle activity during walking tends to be organized in bursts at specific moments of the gait cycle (Figure 1A) to perform specific functions dictated by the biomechanics of bipedal walking (Winter, 1989; Zajac et al., 2003; Lacquaniti et al., 2012). For instance, in early stance hip and knee extensors contribute to weight acceptance at heel contact (Figure 1B). Ankle plantar flexors provide body support and forward propulsion in late stance while ankle dorsiflexors and hip flexors contribute to foot lift-off in early- to mid-swing. Simultaneously, erector spinae muscles activate at this time to stabilize the trunk. In late swing, hamstrings decelerate the leg in preparation for heel contact and then stabilize the pelvis. Throughout the entire step cycle, adductor muscles contribute to the control of medio-lateral accelerations of the center of body mass. However, it is worth noting that most leg muscles that are involved in the control of forward progression in the sagittal plane have a noticeable lateral component of force production and thus are also involved in the control of motion in non-sagittal planes. Even though there is a relationship between the neural and biomechanical control of the gait cycle, evidentially the system is much more complex due to the dynamic coupling of multiple body segments (e.g., Zajac et al., 2003).

Whereas this “invariant” picture of muscle activation (Figure 1A) has been documented in healthy subjects, there are also variant features of muscle activity depending on the context and differences between individuals. If one aims at reactivating the “normal” motor patterns in patients and extrapolating them to any walking condition, this may not be of benefit to the patient due to the specific pathology or individual differences that occur in pathological as well as healthy subjects. Muscle activity in healthy subjects may show very non-linear changes in both amplitude and temporal envelope, e.g., with changing the speed or body weight support even while kinematics patterns remain similar (Ivanenko et al., 2002; Lacquaniti et al., 2002). For instance, the amplitude of EMG activity of “anatomical” synergists may diverge remarkably in these conditions: lateral and medial gastrocnemius muscles at different walking speeds (Huang and Ferris, 2012), soleus and gastrocnemius muscles at different levels of limb loading (McGowan et al., 2010). With body weight unloading (Ivanenko et al., 2002), most muscles (e.g., gluteus maximus and distal leg extensors) decrease their activity, while other muscles demonstrate a “paradoxical” increment of activation (e.g., quadriceps) or considerable changes in the activation waveforms (hamstring muscles). In addition, muscle activity patterns are shaped by the direction of progression (e.g., forward vs. backward, Grasso et al., 1998, or walking along a curved path, Courtine et al., 2006). In particular, such studies suggest that a comparison of normal and pathological gait should be preferably performed in the same stepping conditions.

There is also notable inter-individual variability in muscle activity during walking (Winter and Yack, 1987). The most variable patterns are observed in the proximal and bi-articular muscles especially at lower walking speeds. For example, quadriceps activity is virtually silent in some subjects at low speeds (<4 km/h), whereas it is still present in others (Ivanenko et al., 2002). Finally, notable systematic changes in the EMG activity during walking occur with age, e.g., co-contraction of leg muscles in infants (Forssberg, 1985; Teulier et al., 2012; Ivanenko et al., 2013b) and widening of EMG patterns in elderly (Monaco et al., 2010).

Nevertheless, a number of recent studies using statistical analyses of EMG suggest that the nervous system may adopt a relatively simple control strategy (e.g., Ivanenko et al., 2005; Cappellini et al., 2006; Clark et al., 2010; McGowan et al., 2010; Monaco et al., 2010). Using pattern recognition mathematics, both the stereotypical activation patterns and across-stride variability of these patterns can be accounted for by combining and scaling a small set of basic activation components. Such tendency for a few basic patterns to account for about 90% of variance indicates that leg muscles tend to group their activity in order to perform specific biomechanical functions during gait.

While intuitively one would expect some changes in the EMG activity in patients, the present question is whether the basic modular patterns or the functional grouping of muscles are conserved in pathological participants. Below we summarize various examples of motor patterns in patients with both peripheral and central lesions. The main focus is placed on the studies that analyzed multi-muscle EMG patterns.

## Locomotor patterns in peripheral lesions

Compensatory strategies for plantarflexor weakness or after below-knee amputation represent an important example of gait adaptation. The human bipedal gait and heel-to-toe rolling pattern are unique (Bramble and Lieberman, 2004) and require a specific inter-segmental coordination (Lacquaniti et al., 2002), balance control and walking experience for acquisition of plantigrade gait at the beginning of independent walking (Forssberg, 1985; Ivanenko et al., 2007). Plantarflexor muscles are an important muscle group that regulates the gait speed, compensates for body weight and provides the vertical and horizontal (anterior-posterior shear) forces during the push-off phase. Weakness of the plantarflexors is considered as one of the limiting factors that prevents humans from walking at faster speeds (Nadeau et al., 1999; Brunner and Romkes, 2008).

In addition to the development of extensor forces in the distal antigravity muscles, there is an important sensory feedback from these muscles and from numerous foot receptors. Peripheral neuropathy and aging may result in muscle weakness and substantial impairments of sensory feedback and balance control (Nardone et al., 2000, 2006; Nardone and Schieppati, 2004; Mazzaro et al., 2005). For instance in older adults, ankle plantarflexor work remains relatively constant at increasing speeds, in contrast to the systematic increase in ankle work output with walking speed in young adults (Winter et al., 1990; Judge et al., 1996).

### Patients with large fiber peripheral neuropathy

Weakness of distal extensors in patients with large-fiber neuropathy can be observed after acute nerve compression in the sciatic notch associated with a reduced level of motor and sensory function. After sciatic nerve compression there may be a loss of reflexes, movement skills, sensation in the affected area, and atrophy of the affected muscles can occur (Hagiwara et al., 2003). Sciatica commonly refers to pain that radiates along the sciatic nerve and is typically felt in the back of the leg and possibly to the foot, and is one of the most common forms of pain caused by compression of the spinal nerves.

**Figure 2:**
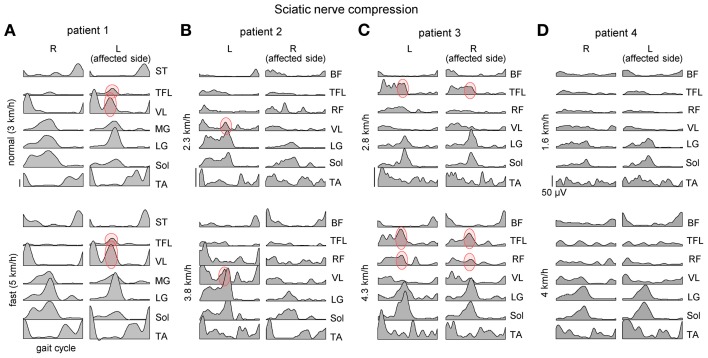
**Ensemble-averaged bilateral EMG activity of leg muscles during overground walking at slow and fast speeds in four patients (panels **A–D**) with unilateral sciatic nerve compression.** Note an “atypical” activation of proximal extensors during late stance (marked in red) and its variability across patients and depending on the affected side.

We analyzed adaptations of gait patterns at different walking speeds in four patients with a unilateral large-fiber neuropathy of S1 innervation resulting from acute nerve compression in the sciatic notch. Plantarflexor weakness on the affected side was evidenced by subjective difficulty to lift and support the body weight on the forefoot region (forefoot-standing) by plantarflexing the ankle joint during standing. Reflexes and sensory thresholds were all normal in the contralateral leg. Figure 2 illustrates EMG patterns in these patients during walking and slow and fast speeds. After acute nerve compression in the sciatic notch, the patients walked somewhat slower (even their self-selected fast speed was <5 km/h) than healthy individuals. The EMG patterns differed from healthy individuals walking at the same speeds (Figure 1A) and were variable between the patients as well as between left and right leg muscle activities (Figure 2). A part of these differences may originate from slightly different EMG electrode placements and/or skin impedance. Nevertheless, we found an interesting cooperation of distal and proximal extensors (Figure 2, marked in red) and discuss its general functional significance for bipedal gait adaptations (Dickey and Winter, 1992; Beres-Jones and Harkema, 2004; Nene et al., 2004; Ivanenko et al., 2008, 2013a).

**Figure 3:**
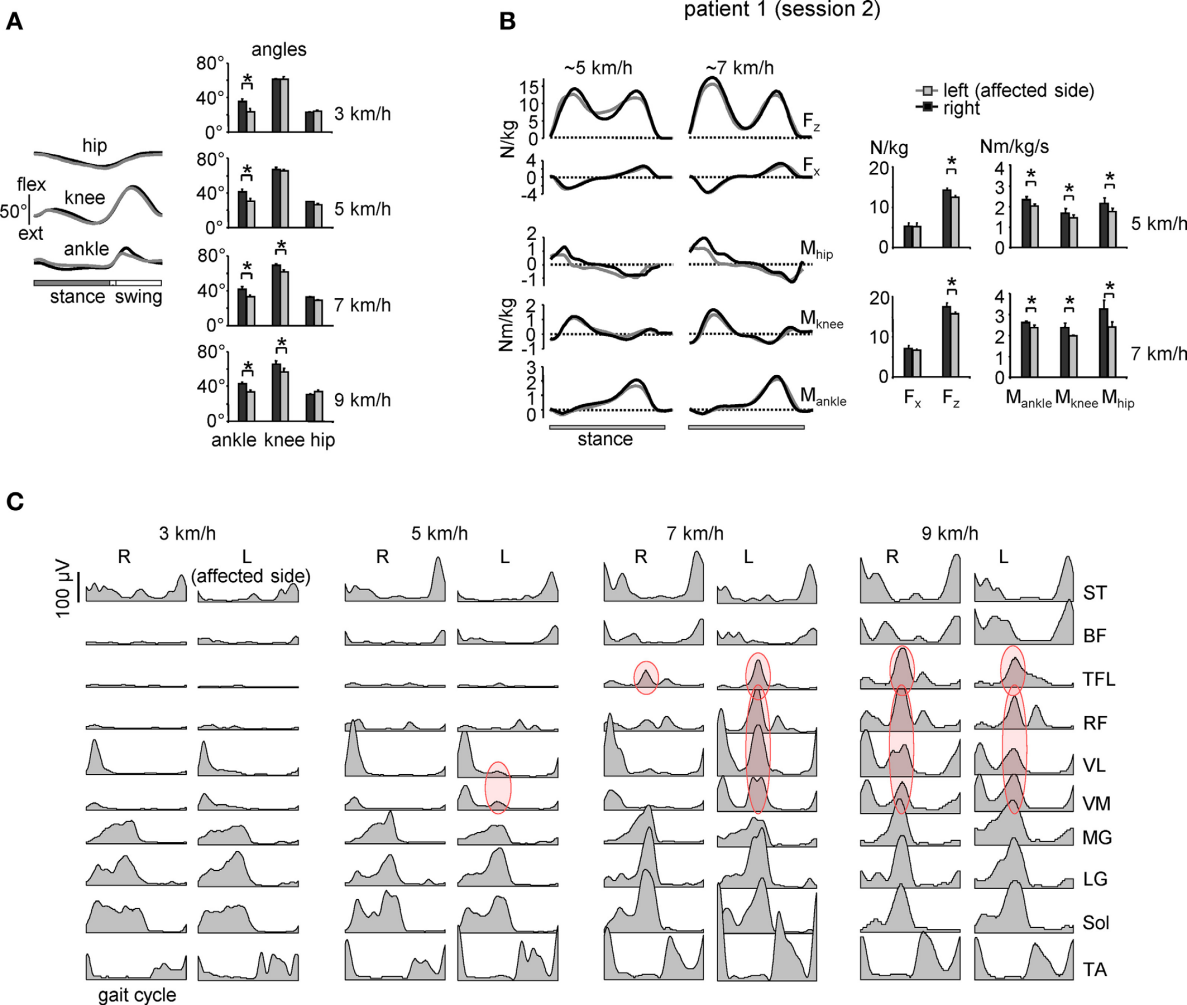
**Motor patterns in a patient with sciatic nerve compression [the same patient as in Figure 2A but recorded 1 year later (session 2)]. (A)** ensemble-averaged (across 12 consecutive steps) joint angular displacements (left panel, mean ± *SD*) and amplitudes of angular joint motion (right panel) during walking at 3 km/h. Asterisks denote significant differences. Note significantly smaller distal joint oscillations on the affected (left) side. **(B)** ensemble-averaged (*n* = 5 steps) vertical (*F*_*z*_) and horizontal anterior-posterior (*F*_*x*_) ground reaction forces, and ankle, knee and hip joint moments of force normalized to the patient's weight during overground walking at ~5 and 7 km/h in session 2 (left panels). The patterns are plotted vs. normalized stance. On the right—peak-to-peak amplitudes. **(C)** Ensemble-averaged bilateral EMG activity of leg muscles during walking on a treadmill at different speeds. In session 1 (Figure 2A), the patient could walk only at speeds up to 5 km/h due to plantarflexor weakness, while in session 2 we recorded walking in a wide range of speeds (3–9 km/h). Adapted from Ivanenko et al. (2013a). Note a prominent burst of activity (marked in red) in the proximal extensors during late stance on the affected (left) side at low speeds in the session 1 (Figure 2A) and only at higher speeds (>5 km/h) in session 2. Furthermore, at 9 km/h the “atypical” burst of activity was present in both legs, as in healthy subjects (see Figure 1A).

The “atypical” burst of activity in the proximal leg muscle was more prominent in patient 1 (Figure 2) on the affected side, while it could be observed also in other patients on the contralateral side. To understand better it's link to the kinematics and kinetics of gait, we recorded patient 1 again during overgound (Figure 3B) and treadmill walking (Figures 3A,C). The second time (1 year later, session 2) the patient had recovered all reflexes and reduced sensation was limited to the lateral, plantar surface of the foot. The patient was now able to fully support his body weight on the left leg during “forefoot standing,” although some weakness still remained. The most prominent decrements in angular oscillations and angular velocities were observed in the ankle joint motion (Figure 3A). In the knee joint, angular motion asymmetry was significant only at higher speeds (7 and 9 km/h). When the plantarflexor strength and/or reflexes were compromised in one leg, the primary compensatory mechanism was an increase in activity of proximal extensor muscles (VL, RF, VM, TFL evidenced by red circles in Figure 2) during late stance. This compensatory effect was observed at all recorded walking speeds in patient 1 in session 1 (Figure 2A) but only at the higher speeds (>5 km/h) in session 2 (Figure 3C) which required greater propulsion forces. This suggests a possible link to the extent of the weakness in the ankle extensors.

Healthy volunteers typically do not show activity in the proximal extensor muscles during late stance in normal walking (Nilsson et al., 1985; Winter, 1989; Prilutsky and Gregor, 2001; Nene et al., 2004; Cappellini et al., 2006). However, the atypical proximal burst was present in all healthy subjects at high, non-preferred walking speeds (Figure 1A, right panel). The proximal muscles involved (RF, VM, VL, SART, Gmed, TFL) were activated synchronously with the distal extensors (MG, LG, SOL) as was observed for the patient. At the higher speeds, the muscle gain, or force produced for a given level of activation, may be lower due to the muscle force-velocity relationship (Neptune and Sasaki, 2005), so the force produced by ankle extensors alone may be insufficient to initiate lift-off or to provide appropriate limb stiffness. The proximal activation may be recruited to compensate by supplying additional extensor torque and stiffness.

Similarities can also be noted between this type of adaptation and unilateral acute pathologies such as hemiplegia (Knutsson and Richards, 1979), below-knee amputation (Winter and Sienko, 1988) or unilateral ischemic block of distal leg muscles in healthy subjects (Dickey and Winter, 1992). Co-activation of distal and proximal extensors during stance in each leg was also observed in the clinically incomplete spinal cord injury individuals during weight-bearing treadmill stepping (Beres-Jones and Harkema, 2004, see also below) and in healthy adults during walking on a slippery surface (Cappellini et al., 2010). Such similarity indicates that this kind of adaptation seems not to depend on the location of the lesion of the neuronal system but may be related to the activation of existing basic activation patterns or muscle synergies.

Nevertheless, despite a potential broad-spectrum functional significance of this compensatory response to plantarflexor weakness, its biomechanical nature remains puzzling. One would perhaps expect the cooperation of distal and proximal extensors during knee-flexed locomotion, e.g., as it happens during digitigrade gait requiring a significant positive (anti-gravity) knee torque during stance. However, in our case (Figure 3B) there was no generation of positive knee moments of force in response to extra activation of knee extensors, likely reflecting dynamic coupling between body segments (Zajac et al., 2003). Moreover, biomechanical simulations of the compensatory strategies in response to muscle weakness do not seem to predict the appearance of “atypical” burst of activity in the proximal extensors (Goldberg and Neptune, 2007). Perhaps a complete consideration of all factors affecting gait optimization is necessary, including a 3D rather than 2D musculoskeletal gait model (e.g., Gmed, TFL, and SART may generate a noticeable trunk torsion or lateral force component, Dostal et al., 1986) and taking into account the mechanisms that regulate leg stiffness during walking (Fiolkowski et al., 2005).

Whatever the exact biomechanical reasons for the observed phenomenon (Figure 3), it is important to emphasize that the timing of the reaction in the proximal muscles corresponded to the timing of the calf muscle activation. This supports the idea of a temporal architecture of the locomotor program linked to specific kinematic events (Ivanenko et al., 2005, 2006; Giszter et al., 2007; McGowan et al., 2010) or critical points in the processing of sensory information (Saltiel and Rossignol, 2004) in the gait cycle.

### Transtibial amputees

Various efforts have been made to restore the normal EMG patterns in patients, presumably by reactivating the CPG (central pattern generator) circuitry or more directly by functional electrical stimulation (FES) (Thrasher et al., 2006; Solopova et al., 2011) or when implementing myoelectric control of powered limb prostheses in amputees (Au et al., 2008; Huang et al., 2011). For instance, transtibial amputees can learn to volitionally activate residual leg muscles (Au et al., 2008; Ha et al., 2011; Hargrove et al., 2011) that can be used for movement intent recognition in the myoelectric control of powered limb prostheses. However, such strategies and their underlying hypotheses are often based on the assumption that the motor patterns are relatively invariant across different walking conditions, for instance, when using FES for gait rehabilitation (Thrasher et al., 2006). Below we consider a reorganization of EMG activity in transtibial amputees.

Below-knee amputation represents a severe damage of the neuromuscular apparatus of the leg and impaired sensory feedback. As a result, the EMG activity in amputees may be compromised by these factors. Indeed, below-knee amputation may result in the EMG patterns different from those in healthy subjects. For instance, co-activation of distal and proximal extensors during stance, similar to that described in the previous section (Figures 2, 3C), was also observed in below-knee amputees (Winter and Sienko, 1988).

**Figure 4:**
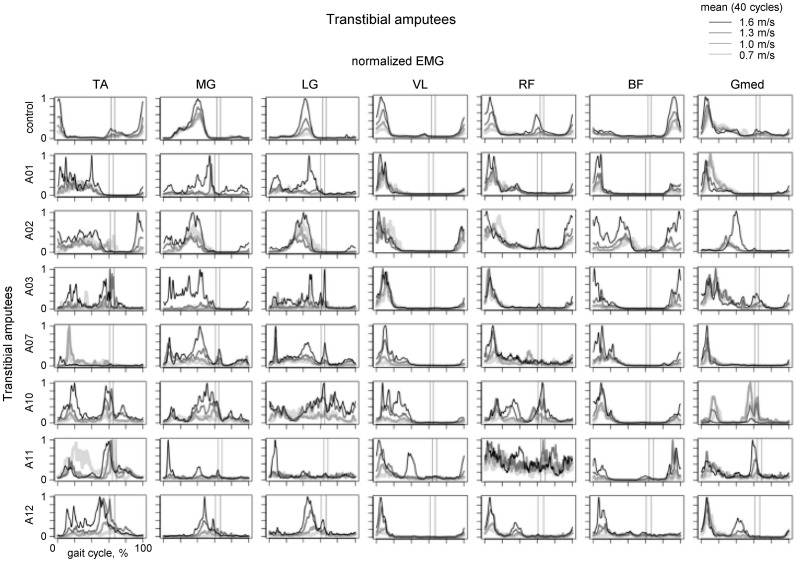
**Ensemble-averaged EMG activity of leg muscles during walking at different speeds in the control group (upper panels) and seven amputee subjects.** Adapted from Huang and Ferris (2012) with permission of the authors. Vertical lines show average toe-off events for the fastest and slowest walking speeds.

Of particular interest is residual lower leg muscle activation following such an amputation. For instance, if the CPG output were relatively fixed (e.g., providing an alternating activity of flexors and extensors, Zehr, 2005) one would not expect major changes in residual muscle activation profiles. Figure 4 illustrates the results of a recent study on multi-muscle EMG activity in both proximal and residual leg muscles during walking in transtibial amputees (Huang and Ferris, 2012). In the upper leg muscles, the data showed that amputee subjects had greater inter-subject variability in their biceps femoris and gluteus medius muscle activation profiles compared to control subjects during walking, as well as a different BF activation profile shape (Figure 4, right panels). Amputee subjects also demonstrated reliable muscle recruitment signals from residual lower leg muscles recorded within the prosthetic socket during walking. However, the grouping of muscles activated together differed from that in controls (see, for instance, “atypical” co-activation of Gmed, BF and MG in the A02 subject or TA, BF and Gmed in the A10 subject). Overall, muscle activation profile variability was higher for amputee subjects than for control subjects (Huang and Ferris, 2012). Nevertheless, it is interesting to note that muscle recruitment signals in amputees tended to be locked to particular phases of the gait cycle (Figure 4).

## Locomotor patterns in central lesions

Contrary to impairments in the peripheral sensory feedback or neuromuscular apparatus, nervous system lesions may essentially affect central controllers and thus provide some insights into the spatiotemporal organization of neural circuitry. In particular, if muscle modules are indeed mechanisms by which task level biomechanical goals are implemented, one would expect that impairments to the neural control of such modules would directly result in impaired biomechanical outputs (Cheung et al., 2009; Ivanenko et al., 2009; Clark et al., 2010). In addition, lesion at different levels of the neuraxis could differentially affect locomotor control. Adaptation of gait after cortical, subcortical or spinal cord damage might thus represent the experimental field in which one might test such hypotheses and the therapeutic relevance of different interventions.

### Stroke patients

**Figure 5:**
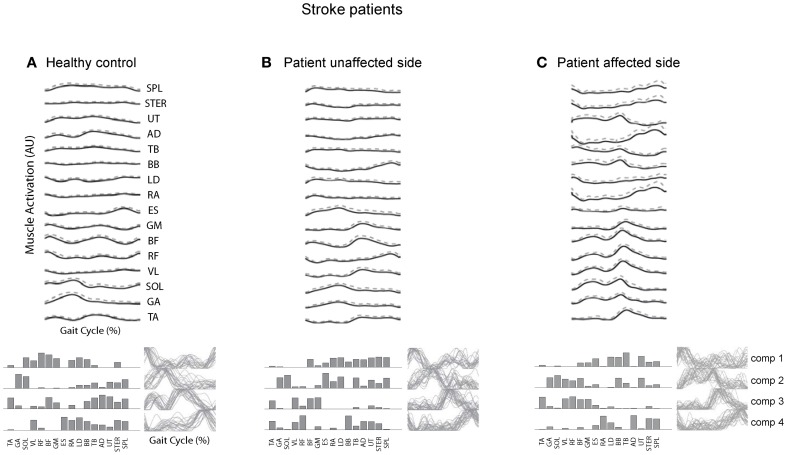
**Surface electromyogram (means and *SD*), motor modules (bottom, left), and activation signals (bottom, right) for a representative healthy control subject (A) and for the unaffected (B) and affected side of a stroke patient. (C)** Adapted from Gizzi et al. (2011).

Post-stroke locomotor impairments are often associated with abnormal spatiotemporal patterns of muscle coordination (Knutsson and Richards, 1979; De Quervain et al., 1996; Mulroy et al., 2003; Den Otter et al., 2007). Furthermore, impaired locomotor coordination in post-stroke may be accompanied by fewer modules (Clark et al., 2010; Safavynia et al., 2011), though in a recent study Gizzi et al. (2011) have argued that impulses of activation rather than muscle synergies are preserved in the locomotion of subacute stroke patients (Figure 5). The discrepancies could be accounted for by a different set of recorded muscles or different populations of patients. The authors of both studies hypothesized that identification of motor modules may lead to new insight into how nervous system injury may alter the organization of motor modules and their biomechanical outputs. Furthermore, entraining appropriate motor modules can be of a major importance for neurorehabilitation of gait in these patients since many of them develop an abnormal stereotype of movement during walking, which is difficult to correct.

A similar conclusion has been reached in recent studies on the upper limb control (Cheung et al., 2009, 2012). All patients studied suffered from a mostly unilateral cortical and subcortical lesion resulting from either an ischemic or a hemorrhagic stroke. The robustness of muscle synergies observed in that study supports the notion that descending cortical signals represent neural drives that select, activate, and flexibly combine muscle synergies specified by networks in the spinal cord and/or brainstem and suggest an approach to stroke rehabilitation by focusing on those synergies with altered activations after stroke. Nevertheless, despite higher variability in muscle activation patterns, all these studies suggest a modular organization of muscle coordination underlying motor control in both healthy and post-stroke subjects.

In fact, one of the effective approaches to gait rehabilitation after stroke consists in using step synchronized FES of leg muscles (Yan et al., 2005; Tong et al., 2006; Ferrante et al., 2008). FES has been shown to be an effective tool for muscle strength augmentation, increase in the range of motion in joints and improvements in walking in neurological patients (Popovic et al., 2009). FES is delivered in reference to the timing of natural muscle excitation during movement and it provides additional sensory reinforcement which ultimately improves learning. As a result, the locomotor centers or networks are excited or released from inhibition in phase with their expected activity and thus may be accessible for correction or stimulating effects (Yan et al., 2005; Tong et al., 2006; Popovic et al., 2009; Solopova et al., 2011). Thus, this approach takes advantage of the spatiotemporal architecture of the locomotor program and increases the patient's functional abilities and the effectiveness of rehabilitation.

### Spinal cord injury

**Figure 6:**
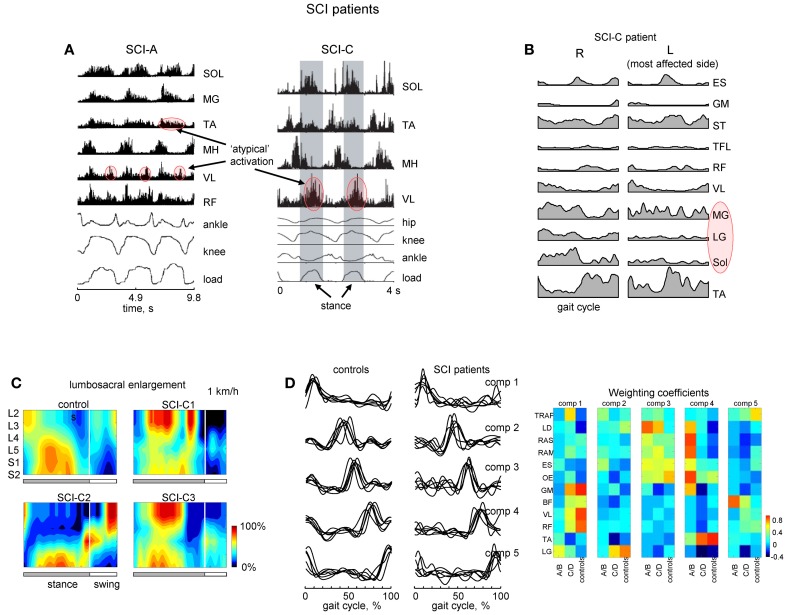
**Motor patterns in SCI patients. (A)** An example of weight-bearing stepping in a clinically complete (at 0.22 m/s, left panel) and incomplete (at 0.89 m/s, right panel) SCI individuals [modified from Beres-Jones and Harkema (2004) and Maegele et al. (2002) with permission of the authors]. The stance phase in the right panel is evidenced by the elevation in the ground reaction force trace and indicated by the shaded region. MH, medial hamstring; load, vertical ground reaction force. **(B)** Ensemble-averaged (across 5 strides) EMG patterns in the SCI-C patient during walking at a natural speed (~3.1 km/h). Note variable and weaker muscle activity on the most affected side (marked in red). **(C)** Examples of spatiotemporal patterns of α-motorneuron activity in the lumbosacral enlargement in controls and three SCI-C patients during walking on a treadmill at 1 km/h. Output pattern for each segment was reconstructed by mapping the recorded EMG waveforms (normalized method, see Ivanenko et al., 2006) onto the known charts of segmental localization. White vertical lines denote stance-to-swing transition time. **(D)** Time course of the temporal components in controls and patients for stepping at 2 km/h, 0–75% body weight support. The components extracted by factor analysis from individual subjects. Right panel illustrates weighting coefficients of the temporal components in individual activity patterns of 12 muscles for all groups of subjects in a color coded scale. Adapted from Ivanenko et al. (2003). Note similar basic EMG components in controls and patients as opposed to quite different EMG patterns and weighting coefficients.

Flexibility and adaptability of locomotor patterns are evident from monitoring and analyzing the spatiotemporal spinal segmental output after spinal cord injury. For instance, in motor incomplete paraplegics who recovered independent control of their limbs, an additional activation burst is present in the lumbosacral enlargement at full loading (Figures 6A,C). The presence of this burst is related to abnormal activation of the quadriceps muscle during this time. Patients can be trained to step with body weight support unassisted, but they use activity patterns in individual muscles that were often different from healthy individuals.

A number of clinical trials have suggested the possible beneficial effects of locomotor training in SCI patients (Edgerton and Roy, 2012). In patients with severe SCI disorders, initial training is performed while being supported by a harness or with their body partially unloaded. As well, assistance of leg movements by the therapist (or robotics) may be required. These patients frequently show EMG patterns different from those of healthy individuals suggesting that human spinal cord can interpret differently loading- or velocity-dependent sensory input during stepping (Beres-Jones and Harkema, 2004). One method used to study such variability involves reconstructing the total output pattern of motoneuron activity of the lumbosacral enlargement of the spinal cord by mapping the recorded EMG waveforms onto the known charts of segmental localization (Ivanenko et al., 2006). Spatiotemporal maps of motoneuron activity are generally different from those of healthy subjects (Figure 6C; Grasso et al., 2004). The legs may also show muscle activity which is not-systematically synchronized with the gait cycle on the most affected side (Figure 6B; marked in red). Although training of stepping in patients with body weight support can be facilitated in a laboratory setting, often new coordinative strategies appear. Training can be utilized in order for patients to relearn foot kinematics of healthy individuals, but the muscle activation patterns used to generate these kinematics differ from that of a healthy group (Pepin et al., 2003; Grasso et al., 2004). While most incomplete paraplegics can recover independent control of leg muscles sufficient to propel the limbs in swing and to support body weight in stance, complete paraplegics, were unable to and they typically used their arms and body to assist the leg movements. SCI patients largely relied on proximal and axial muscles to lift the foot and to project the limb forward.

It is though that spinal lesions may trigger plasticity including modified synaptic strengths, sprouting and anatomical development of new circuits as well as plasticity of unlesioned descending pathways, including both subcortical and cortical motor areas. Stepping may also depend more heavily on cortical (and voluntary) control after severe spinal lesions (Van den Brand et al., 2012) than it does in healthy subjects, where locomotion may be more automatic. The spinal cord itself does also contribute to the proposed adaptation mechanisms. Indeed, experiments on both animals and SCI patients suggest that the spinal cord is capable of adaptive locomotor plasticity with training after spinal lesion (Hodgson et al., 1994; Belanger et al., 1996; Heng and de Leon, 2007) or after peripheral motor nerve lesions (Bouyer et al., 2001).

Modular pattern generator elements (or burst synergies), nevertheless, tend to be conserved after spinal cord injury (Fox et al., 2013; Giszter and Hart, 2013). Our previous work in SCI patients (Ivanenko et al., 2003) has shown a similar set of temporal components from EMG activity (Figure 6D). In addition, muscles both rostral and caudal to the lesions could be strongly weighted on a given component. However, some patients do exhibit a smaller number of basic components during walking (Ivanenko et al., 2003; Hayes et al., 2011; Fox et al., 2013) although these results may be dependent on the number and selection of muscles recorded during stepping. Nevertheless, similar activation timings in SCI patients (Figure 6D) may be ultimately related to the global kinematic goal (a motor equivalent solution; Grasso et al., 2004) or the necessity to apply forces at particular phases of the gait cycle (Lacquaniti et al., 2012). These data highlight the importance of understanding the modular structure of motor behaviors to best provide principled therapies after central nervous system lesions (Giszter and Hart, 2013).

## Concluding remarks

Taken together, the data support the idea of plasticity and distributed networks for controlling human locomotion (Scivoletto et al., 2007). Tens of muscles participate in the control of limb and body movements during locomotion, and redundancy in the neuromuscular system is an essential element of gait adaptability (Winter, 1989; Cai et al., 2006; Noble and Prentice, 2006; Ivanenko et al., 2009; Molinari, 2009; Duysens et al., 2013). Indeed, experimental studies performed on individuals with well-identified pathologies have demonstrated distinct adaptations. Due to muscle redundancy, various neuromotor strategies may exist to compensate for decreased muscle strength and joint stiffness (Grasso et al., 2004; Goldberg and Neptune, 2007; Ivanenko et al., 2009; Gordon et al., 2013).

A modular motor organization may be needed to solve the degrees of freedom problem in biological motor control (Giszter et al., 2010). Nevertheless, there are still many open questions related to the choice of appropriate modules, their task dependence, influence of sensory input and adaptation to the malfunctioning of neural networks in the case of different gait pathologies. While many studies succeeded in a decomposition of motor patterns into a few “motor modules,” nevertheless, the way in which the central nervous system combines them together, how and where the weighting coefficients are encoded are not understood. Often it is difficult to distinguish what primarily comes from pathology and what comes from compensatory mechanisms. We suggest that many adaptive features in various neurological disorders are likely compensatory, including modified EMG patterns during walking. In view of task dependence of muscle synergies, it would be interesting also to compare them in different behaviors and examine whether plasticity in muscle patterns originates from sharing these common modules or by creating new muscle synergies.

An impulsive (burst-like) controller made of a low-dimensional set of time-delayed excitation pulses has been also thoroughly considered in a simulation study from the biomechanical viewpoint (Sartori et al., 2013). In particular, simulated gait motions based on a few modular activation patterns were successfully produced (see also Neptune et al., 2009; Allen and Neptune, 2012; Allen et al., 2013). Once calibrated, the musculoskeletal model could work in open-loop, approximating joint moments over multiple degrees of freedom using only the recorded kinematics and the internal impulsive controller. The accuracy of estimation of the joint torques was comparable when using the low-dimensional activation signals (Sartori et al., 2013). This approach has substantial implications for the design of human machine interfaces for prosthetic and orthotic devices.

Uncovering a common underlying neural framework for the modular control of human locomotion and its development represent an interesting avenue for the future work. Motor primitives may reflect in a way how the nervous system develops, by building up or modifying modules as it matures. Some functional units are likely inborn, others may develop later or be dependent on individual body size/proportions or experience (Dominici et al., 2011). Such investigations may have important implications related to the construction of gait rehabilitation technology.

### Conflict of interest statement

The authors declare that the research was conducted in the absence of any commercial or financial relationships that could be construed as a potential conflict of interest.
